# Efficacy and safety of Jiu-Wei-Xi-Feng granules for treating tic disorders in children: study protocol for a randomized controlled equivalence trial

**DOI:** 10.1186/s13063-022-06802-y

**Published:** 2022-10-22

**Authors:** Sheng-xuan Guo, Rui-ben Li, Si-yuan Hu, Qiu-han Cai, Cheng-liang Zhong, Rui-min Hao

**Affiliations:** 1 Department of Clinical Trial Center, First Teaching Hospital of Tianjin University of Traditional Chinese Medicine, National Clinical Research Center for Chinese Medicine Acupuncture and Moxibustion, No. 88 Changling Street, Xiqing District, Tianjin, 300193 China; 2 Department of Pediatrics, First Teaching Hospital of Tianjin University of Traditional Chinese Medicine, National Clinical Research Center for Chinese Medicine Acupuncture and Moxibustion, No. 88 Changling Street, Xiqing District, Tianjin, 300193 China; 3 Department of Acupuncture and Moxibustion, First Teaching Hospital of Tianjin University of Traditional Chinese Medicine, National Clinical Research Center for Chinese Medicine Acupuncture and Moxibustion, No. 88 Changling Street, Xiqing District, Tianjin, 300193 China

**Keywords:** Jiu-Wei-xi-Feng granules, Tic disorders, Randomized controlled trial, Equivalence trial, Traditional Chinese medicine

## Abstract

**Background:**

Tic disorders (TD) is a neuropsychiatric disease with twitch as the main manifestation in childhood. Jiu-Wei-Xi-Feng granules has been marketed in China for treating children with TD. As *Long Gu* (Os Draconis) in the composition of this Chinese patent medicine is a rare and expensive medicinal material protected by the Chinese government, therefore, we consider replacing it with *Mu Li* (Concha Ostreae) that has the same effect and is cheaper. This study is designed to evaluate the clinical equivalence between Jiu-Wei-Xi-Feng granules (*Os Draconis* replaced by *Concha Ostreae*) (JWXFD) and Jiu-Wei-Xi-Feng granules (original formula) (JWXFO) in children with TD (consumption of renal yin and liver wind stirring up internally syndrome).

**Methods/design:**

This is a multicenter, randomized, double-blind, equivalence trial comparing the efficacy and safety of JWXFD and JWXFO in treating Children with tic disorders (consumption of renal yin and liver wind stirring up internally syndrome). A total of 288 patients will be recruited and randomly assigned to two groups in a 1:1 ratio. The treatment course is 6 weeks, with a 2 weeks follow-up. The primary outcome is the mean change value from baseline to 6th week by the Yale Global Tic Severity Scale total tic score (YGTSS-TTS). Secondary outcomes include total effective rate of tic, Yale Global Tic Severity Scale (YGTSS) scores and its factor scores (the degree of motor tics, phonic tics and social function damage), Clinical Global Impression-Severity scale, and TCM syndrome efficacy.

**Discussion:**

The design of this study refers to a large number of similar research design points, and asked for opinions of peer experts, and finally reached a consensus. This trial will provide high-quality evidence on the clinical equivalence between JWXFD and JWXFO and provide a basis for the marketing of JWXFD.

**Trial registration:**

ChiCTR2000032312

Registered on 25 April 2020, http://www.chictr.org.cn/showproj.aspx?proj=52630

## Background

Tic disorders (TD) is a neuropsychiatric disorder characterized by involuntary, aimless, rapid, repetitive, and rigid single or multiple muscle motor tics and/or vocal tics in childhood and adolescent [1, 2]. The therapy of tic disorders usually consists of three stages: psychological education, behavior intervention, and drug treatment [3, 4]. Behavioral therapy has become a first-line treatment for TD, but it is limited by the need for professional intervention personnel and the long intervention time. When patients require an active intervention for these symptoms and behavioral therapy is not available, antidopaminergic drugs (tiapride, haloperidol, aripiprazole, tetrabenazine, etc.) and alpha adrenergic receptor agonists (guanfacine, clonidine) should be considered. However, they often bring many adverse effects. Tiapride, which is safe and good, will still have up to 25% sedation or drowsiness and cause the female prolactin level abnormality or amenorrhea and so on [5–7].

The description and management methods of TD-like symptoms first appeared in ancient Chinese medical documents *Inner Canon of Huangdi* written 2000 years ago. Essays on the therapeutic prescription of TD can be found in Key to Therapeutics of Children's Diseases [8], written by Qian Yi of Song Dynasty. As time goes by, TCM has made a great progress in tic disorders, more abundant experience in syndrome differentiation and treatment, and summarized excellent and effective TCM prescriptions [9, 10].Jiu-Wei-Xi-Feng granules, developed by Jiangsu Kanion Pharmaceutical limited company, originated from the clinical experience of treating children's tic disorders with consumption of renal yin and liver wind stirring up internally syndrome. From 2004 to 2009, Jiu-Wei-Xi-Feng granules completed the phase II and phase III clinical trials controlled by tiapride and the supplementary trials controlled by placebo [11–13], and it passed the examination of Center for Drug Evaluation of China. The product was approved for production and marketing on December 31, 2012, by China food and drug administration (CFDA) (approval No.:z20120034). It is composed of *Shu Di Huang* (*Rehmanniae Radix Preparata*), *Tian Ma* (*Gastrodiae Rhizoma*), *Long Dan* (*Radix Gentianae*), *Gui Jia* (*Carapax et Plastrum Testudinis*), *Gou Teng* (*Ramulus Uncariae Cum Uncis*), *Long Gu* (*Os Draconis*), *Jiang Can* (*Bombyx Batryticatus*), *Qing Meng Shi* (*Lapis Chloriti*), and *Fa Ban Xia* (*Rhizoma Pinelliae Preparatum*)*.*

Regulations on the protection of fossil paleontology, promulgated by the State Council of China in September 2010, stipulate that no unit or individual shall transact key protected fossils without authorization. The *Long Gu* (*Os Draconis*), one of the prescriptions, belongs to first-class key protected fossil of paleontology. In view of the relevant policies and regulations of national *Longgu* protection, in order to save resources, we consider replacing the *Long Gu* (*Os Draconis*) with *Mu Li* (*Concha Ostreae*)*.* Both of them have similar functions in traditional Chinese medicine and basically consistent chemical composition. They often use each other interchangeably in Chinese herbal compound prescription. At the same time, we also studied the pharmacodynamics of Jiu-Wei-Xi-Feng granules (JWXFD) which substitutes *Concha Ostreae* for *Os Draconis* and original Jiu-Wei-Xi-Feng granules (JWXFO) in three animal models. The results showed that both of them could reduce the score of the relevant evaluation indexes of the tics and without statistical difference between groups. In order to further confirm the feasibility of replacing keel with oyster, we submitted an application to the domestic drug administration, which will be further verified through standardized clinical trials.

There are many internationally registered clinical trials on TD in children, most of which are chemical drugs. The overall design of clinical trial control for new drugs such as first-line therapeutic drugs such as aripiprazole and benzoxazines witch is under development is placebo as a control, and the superiority test is used to prove its effectiveness and safety [14, 15]. The clinical trials of new traditional Chinese medicine for children’s TD have gradually matured. In recent years, studies on the treatment of children’s TD with Wu-ling Granules, Ning-dong Granules and other prescription controlled placebos/positive drugs are increasing [16, 17]. The research on the treatment of mental disorders with botanical drugs such as Bacopand and Passion flower has gradually developed, and the overall design is basically the same as that of the clinical trials of chemical drugs [18]. It can be seen that placebo is the main type of control in clinical trials of children’s TD drugs. In this study, the clinical evaluation of Jiu-Wei-Xi-Feng granules after *Mu Li* (Concha Ostreae) replaced *Long Gu* (Os Draconis) has its particularity. First of all, original Jiu-Wei-Xi-Feng granules have been marketed and have good therapeutic effect. Secondly, *Long Gu* (Os Draconis) and *Mu Li* (Concha Ostreae) have similar properties and tastes in traditional Chinese medicine, and their modern chemical analysis components are basically the same, considering that *Mu Li* has mature practical experience in replacing *Long Gu* in clinical application of traditional Chinese medicine and combining with the results of earlier pharmacodynamics research. After careful communication between the research team and the drug regulators, the clinical equivalence test of the new prescription against the original prescription was conducted to evaluate their effectiveness and safety.

## Methods/design

### Study design

**Fig. 1:**
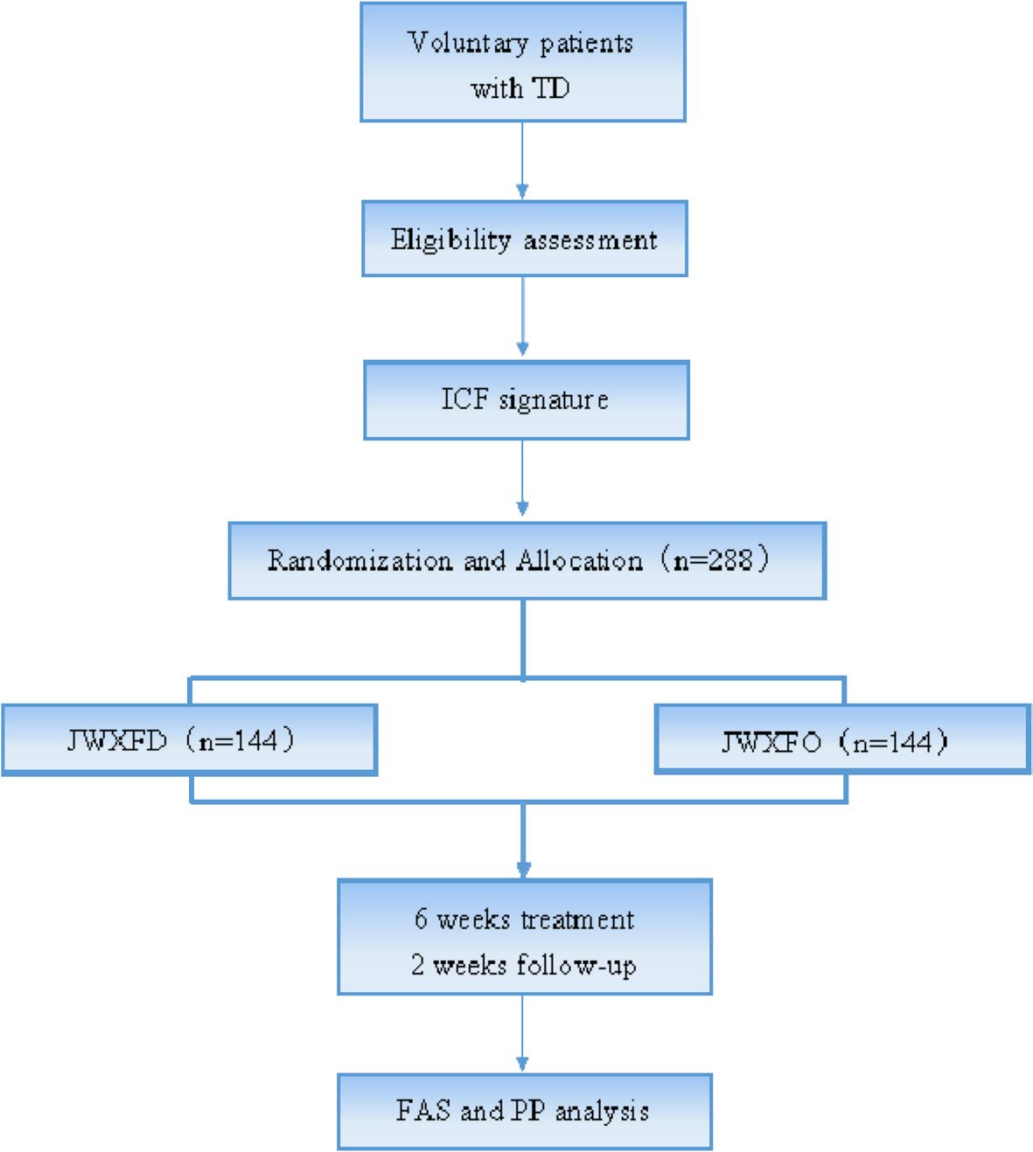
Study flow chart

This study is a multicenter, randomized, prospective, double-blind, and parallel group, equivalence trial comparing the efficacy and safety of JWXFD (*Os Draconis* replaced by *Concha Ostreae*) and JWXFO (original formula) in treating TD in Children. Figure 1 shows the flow chart of the trial.

### Ethics

This study has been authorized by the China Food and Drug Administration (CFDA) (approval number SFDACYZB1805909) and registered in the Chinese Clinical Trial Registry (ChiCTR2000032312). In addition, the trial was approved by the Ethics Committee of The First Teaching Hospital of Tianjin University of Traditional Chinese Medicine (TCM) (approval number (TYLL2019[Y]-017) and the ethics committees of the other twelve hospitals. To ensure the enough participants and research progress, 13 clinical research center in different provinces of China will conduct this trial: First Teaching Hospital of Tianjin University of Traditional Chinese Medicine, The Affiliated Hospital of Shandong University of TCM, Daqing People’s Hospital, Second Affiliated Hospital of Tianjin University of TCM, Wuxi Children’s Hospital, Dongzhimen Hospital Beijing University of Chinese Medicine, Children’s Hospital of Soochow University, The First Affiliated Hospital of Henan University of TCM, The Second Hospital of Hebei Medicine University, Taizhou Hospital of TCM, The First Hospital of Hunan University of Chinese Medicine, First Affiliated Hospital of Heilongjiang University of TCM, and Deyang People’s Hospital. If there is any important modification to the study protocol, it will be submitted to the ethics committee again for review and approval. Each center selects several qualified and experienced doctors as researchers; in terms of experimental recruitment, we also issued electronic recruitment advertisements (WeChat, QQ, microblog and other social software) and posted paper recruitment advertisements (such as exhibition boards, posters, etc.).

Before screening, researchers should explain all the processes and details of the project to guardians and children so that they can fully understand and consider them. Article 20 of the General Principles of the Civil Law of the People’s Republic of China stipulates “Minors under the age of eight are persons without capacity for civil conduct, and their agents ad litem shall act as agents for civil juristic acts.” All children ≥ 8 years old and their guardians should sign the informed consent form before entering the study, and children under 8 years old should only be signed by their guardians. Researchers should let children realize that participation is completely voluntary, and they have the right to withdraw from the study at any time. If there is any injury or death related to the trial, the sponsor will be responsible for the related treatment costs and financial compensation. After participating in the study, the personal information of the children will be abbreviated to protect their privacy. The data related to the trial process of subjects are only allowed to be viewed by researchers and monitors and will not be used in other studies. The study protocol is based on the SPIRIT list [19]. The results of this study will be submitted to peer-reviewed journals.

### Patient population and setting

#### Diagnostic criteria

**Fig. 2:**
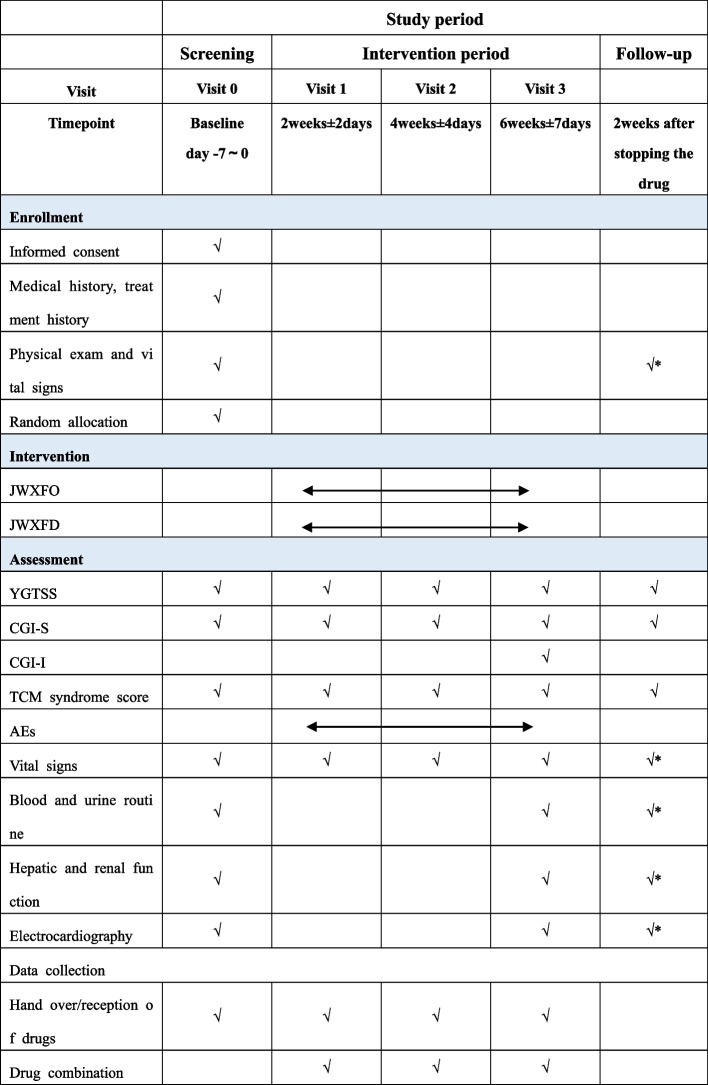
Schedule of study procedures. The symbol √* indicates the following: if there is an AE, the patient needs to be followed up for safety information till they reach the endpoint of event

A diagnosis of the TD in Western medicine is established according to DSM-5 [20]. Standard of TCM syndrome differentiation is based on *Pediatrics of TCM* (*Second Edition* [21])*.* The TCM diagnostic differentiation criteria for CEYLWSS are listed in Fig. 2. Patients should have at least one primary symptom and at least two of the secondary symptoms in order to confirm the syndrome differentiation, as well as refer to tongue and pulse.

According to *Diagnosis and treatment of childhood tic disorders experts consensus* (*2017*) [1] published by The Neurology Group of Chinese Pediatric Society of Chinese Medical Association, the criteria for the severity of TD based on Yale Global Tic Severity Scale (YGTSS) are as follows:

1. Mild: YGTSS total scores < 25
2. Moderate: YGTSS total scores between 25 and 50
3. Severe: YGTSS total scores > 50

#### Inclusion criteria

1. Meeting the diagnosis criteria of TD of mild or moderate based on DSM-5 (Diagnostic and Statistical Manual of Mental Disorders, Fifth Edition) [20] and confirmed the TCM syndrome pattern criteria of consumption of renal yin and liver wind stirring up internally syndrome (CEYLWSS)
2. Aged between 4 and 14
3. The participants’ TD last for more than a year and had not used any related therapeutic drugs for TD before 2–4 weeks enrolled (> six half-lives plus 1 week for the related drugs)
4. Parents or other legal guardians of the children sign informed consent

#### Exclusion criteria

1. Participant with transient tic disorder (TTD), other specific tics or non-specific tics, severe TD, and refractory TD (the TD patients who have been treated with sufficient anti-TD drugs such as tiapride hydrochloride and aripiprazole for more than 1 year without effective and whose course of disease is prolonged [1])
2. Participants’ involuntary movement can be explained by other diseases, such as rheumatic dance, Huntington’s dance, Kayser’s disease, Hammond’s disease, myoclonus, acute dyskinesia, spasm of hysteria, epilepsy and childhood schizophrenia, and drug-induced extrapyramidal diseases
3. Participant combined with other mental disorders such as attention deficit hyperactivity disorder (ADHD), obsessive-compulsive disorder (OCD), learning disorder, and sleeps disorder
4. Participant with severe primary diseases of the heart, liver, kidney, digestion, and hematopoietic system
5. Participant with allergic reaction to composition of experimental drug
6. Participant cannot cooperate or be participating in other drug trials
7. The researcher consider that the participant is not suitable to be enrolled

#### Withdrawal criteria

1. The investigator can decide the patient to quit in case of allergic reaction or serious adverse events
2. After 4 weeks of treatment, participant is worsening conditions (YGTSS total scores > 50)
3. During the study, participant with other diseases, or stopping the experimental drug for more than a week
4. Participant has poor compliance (the compliance is less than 80%), automatically changes the medication or adds the prohibited TCM medicine or western medicine during the study
5. Breaking blindness
6. Participant is found to have seriously violated the inclusion and exclusion standards after randomization
7. Patients have right to withdraw for any reason during the study
8. The subjects did not explicitly claim withdraw from the study, but they do not take the drug and accept follow-up visit

### Randomization and blinding

A total of 288 eligible subjects will be randomly allocated in a 1:1 ratio. Randomized sequences was generated by an independent statistical expert in the Epidemiology Department of First Teaching Hospital of Tianjin University of TCM, with 36 blocks of block size 8 based on the computer software SAS 9.2. Randomized sequences is duplicate, which will be concealed in the sealed envelopes, managed by the project managers of each center and sponsor, who are not involved in the recruitment, intervention, assessment, or statistical analysis. Subjects, investigators, CRC and statisticians were blind. The color and taste of the new prescription (NJWXD) and the original prescription (JWXFO) were basically the same after the assessment of school-age children. Therefore, the method of double-dummy was not adopted in this trial. Emergency envelopes are kept in each study center; the investigator may only open it under the following conditions: patient occurs serious adverse event (SAE) or serious infection and patient’s condition worsened and need for necessary emergency treatment.

### Interventions

All the drugs (JWXFD and JWXFO) of this trial are provided and manufactured by Jiangsu Kanion Pharmaceutical Co., Ltd (Jiangsu, China).JWXFD or JWXFO is 6 g per bag (batch number: 190701 for JWXFD; 190702 for JWXFO). Children 4 to 6 years old receive 1 bag twice a day Bid, while those 7 to 9 years old take 1.5 bags Bid, and those 10 to 14 years old take 2 bags Bid. The drugs of each subject were packed in a separate package for 2 weeks or plus 3 days. The duration of treatment was 6 weeks and the follow-up was 2 weeks. During the trial, the researchers will visit the patients for four times, with an interval of 2 weeks. During the follow-up, the researchers will ask the children about their medication and carry out drug recovery, distribution, and disease assessment. The surface of each package attach a visible label states “Jiu-Wei-Xi-Feng Granules For Clinical Trial Use Only” and other information: national drug approval number, drug number, drug name, package dosage, storage conditions, and drug supplier. All test drugs shall be collected and counted by special personnel, and the compliance shall be calculated. Details of the study schedule are in Fig. 2.

#### Concomitant treatments and forbidden medication

During the study, any other therapy or medication may affect the study outcomes is prohibited, such as tiapride, clonidine, haloperidol, valproate, aripiprazole, risperidone, tetrabenazine, pimozide, fluphenazine, sulpiride, inosine, and with the same effect Chinese medicine is also prohibited. The severity of TD children included in this study is mild to moderate, that is, the YGTSS score is less than 50 points, which conforms to the indications of JWXFO. Therefore, in theory, it will not cause greater harm to patients when other drugs are not applicable during the limited test. In order to protect the children under test, if the children’s condition worsens during the test, they can decide to withdraw according to the change of the condition. In addition, drug intervention is allowed in cases unrelated to TD. Any other treatment during the study will be recorded in the case report form (CRF).

### Outcome measures

#### Primary outcome

The primary outcome is the mean change from baseline to the 6th week in YGTSS total tic score (YGTSS-TTS). YGTSS is a special scale developed by Leckman et al. of Yale University in 1989 to evaluate the severity of Tic disorders. It contains three parts: motor and phonic tics and social function impairment. YGTSS-TTS refers to the integral sum of the first two parts [22].

#### Secondary outcomes

**Fig. 3:**
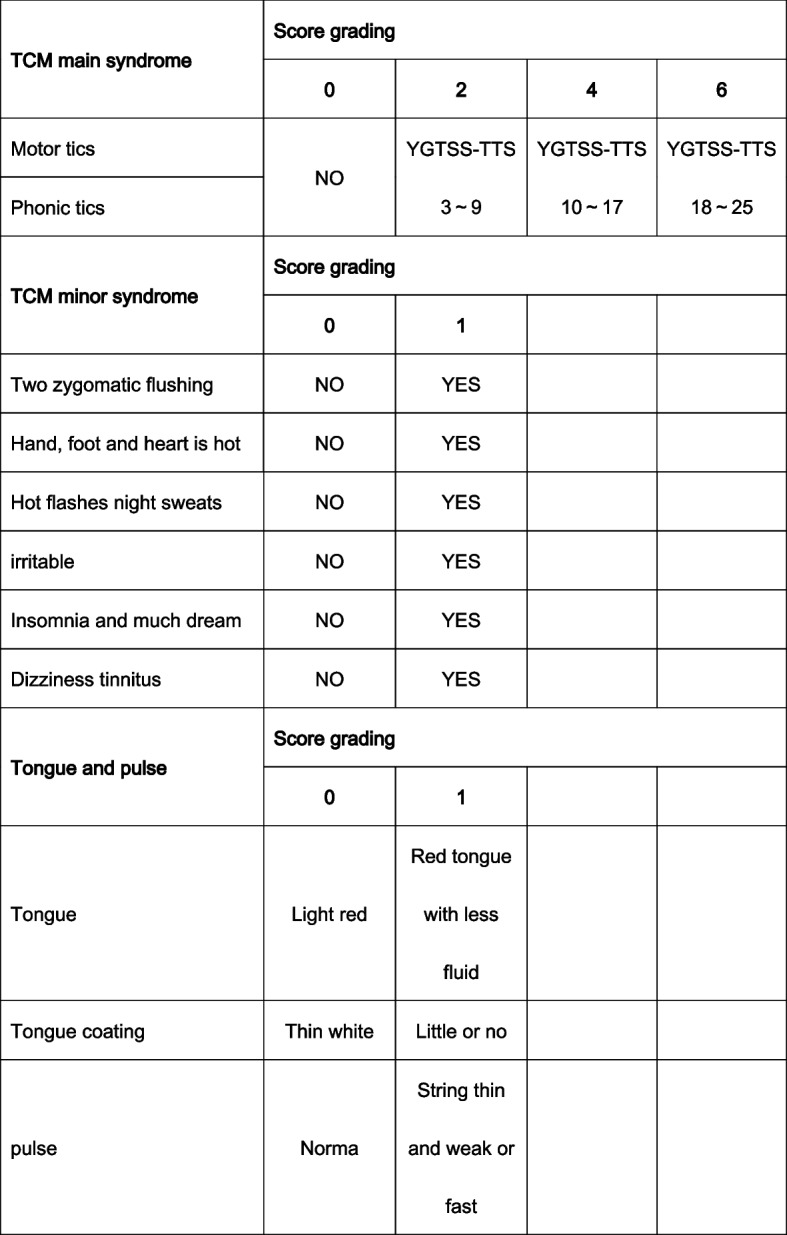
Symptoms and signs scale based on TCM syndromes. For symptoms that cannot be accurately expressed due to the age of the child, fill in NA

Secondary outcomes include total effective rate of tic, YGTSS scores and its factor scores (the degree of motor tics, phonic tics and social function damage), Clinical Global Impression-Severity scale (CGI-S), and TCM syndrome efficacy. Effective rate of tic is judged by the change in YGTSS-TSS score from initial assessment to that at the end of the 6th week. Clinical control is defined as ≥ 75% decrease in the YGTSS-TSS score; obvious improvement as ≥ 50% and < 80% decrease; improvement as ≥ 25% and < 50% decrease, and ineffective as < 25% decrease. Total effective rate of tic = clinical control + obvious improvement + improvement. The CGI-S is used to evaluate the severity of clinical symptoms and change that ranges from 0 (normal) to 7 (extreme) [23, 24]. The “effective” of TCM syndromes is defined as the reduction of the total score of TCM syndromes ≥ 50%. Change in TCM symptom and sign scores based on Fig. 3. The primary symptoms (motor tics and phonic tics) was measured as either none (0), mild (2), moderate (4), or severe (6). The secondary symptoms was measured as either none (0) or being (1). Total TCM scores is equal to total primary symptoms plus total secondary symptoms.

### Safety outcome measures

The primary outcome of safety is the incidence of adverse events. Some physical examinations and laboratory tests will be performed on baseline and after 6 weeks of treatment to assess safety.

The physical examination includes temperature, respiration, blood pressure, and heart rate. Laboratory tests included blood routine, urine routine, liver and renal function, and ECG. Adverse events (AE) will be recorded in CRF; “adverse event record” of CRF includes adverse event occurrence time, end time, duration, severity, measures taken and outcome, and make judgment on the relationship between adverse events and experimental drugs.

### Sample size estimation

The placebo-controlled clinical trial of JWXFO showed that the mean difference of YGTSS-TTS was 6.12(4.12~8.12) and standard deviation was 7.2. We prespecified an equivalence margin of − 3 to 3 points. Assuming a 15% dropout rate, the sample size of 144 per group should provide an approximately 80% power at a statistical level of 0.05.

### Data management, management, and audit

Two clinical research coordinators (CRC) independently entry the data into the online electronic data capture (EDC) based on the CRFs, and the data entry time shall not exceed 1 week after each visit. After this, investigator and data administrator shall audit the data in accordance with the CRFs independently. After the completion of data entry, any data modification in EDC need fill in a reason and the modification record will be left.

Conduct data verification according to the data verification plan formulated by the data administrator. The data verification methods are divided into system verification and manual verification (the system verification is carried out according to the EDC logic verification rules, and the manual verification part is manually implemented by SAS v9.2 programming or data administrator). The questions arising from the data verification shall be answered and returned by the researcher as soon as possible. The data administrator shall modify the data according to the answers of the research doctor/researcher. If necessary, the DRQ can be issued again until all the questions are answered.

The sponsor, researcher, data administrator, and statistician jointly conduct the final audit on the unresolved problems in the data and form a blind audit report. During data review, all cases of data query, drop off and protocol deviation, concomitant drugs, and adverse events were discussed, and the population was divided for statistical analysis. Once an agreement is reached, the database will be locked and no modifications are allowed thereafter. Statistical analysts conduct data analysis in a blind state.

### Statistical analysis

After that, clinical researchers, data managers, and statisticians will hold a data verification conference under the blind state for discussing data process and the partition of data sets including full analysis set (FAS) population, the per-protocol set (PPS) population, and safety set (SS) population. Statistical analysts perform data analysis in a blind state. Efficacy analyses will be conducted according to the full analysis set (FAS) population and the per-protocol set (PPS) population. Safety analyses will be conducted by safety set (SS) population. Missing values of primary outcome will be replaced by the last observation carried forward (LOCF) method. *T* test or non-parametric test will be used for quantitative data, while chi-square test will be used for qualitative data. Cochran-Mantel-Haenszel test or logistic regression model will be used to adjust covariates (center or others). The difference of primary outcome was compared between groups using a two-sided confidence interval approach, a level of equivalence between treatments of ±3 points and a 0.05 significance level. SAS 9.2 will be used for data analysis.

### Quality control and monitoring

To ensure the test quality, each study hospital will have a professional quality manager. All investigators will receive uniform training about standard operating procedures (SOPs) for trial execution. In particular, the investigators involved in the evaluation, we will conduct conformance training on YGTSS scale, CGI-S, and TCM symptom grading scale. CRCs are responsible for contacting subjects regularly to remind them of medication and follow-up. CRA regularly monitors the quality of trial to ensure their authenticity and integrity.

An independent expert committee composed of clinical experts, statisticians, pharmaceutical experts, pharmacology, and toxicology experts was established to monitor and evaluate patient safety and efficacy data blindly. It will also assess whether the participants have received good explanations and appropriate solutions to clinical nursing and safety issues, as well as suggestions for protocol modification, and even suggestions and decisions for terminating the study based on efficacy and safety.

Auditors independent of researchers and sponsors will conduct monthly reviews to systematically review activities and documents related to the study to assess whether the study is conducted in accordance with the study protocol, GCP, and relevant regulatory requirements and whether the test data is recorded in a timely, true, accurate, and complete manner.

## Discussion

TD is a chronic neuropsychiatric disease with a long treatment period. Most of the children with TD had improved symptoms after treatment, while a few cases showed no significant improvement and the symptoms persisted until adulthood or lifelong [25]. The exact etiology and pathogenesis of TD are unclear. It is generally believed that genetic factors and environmental factors work together to cause changes in biological substances in vivo leading to different clinical manifestations and comorbidities. Traditional Chinese medicine holds that the main pathogenesis of TD is consumption of renal yin and liver wind stirring up internally [26]. The treatment of TD is mainly behavioral therapy and drug therapy. When the severity of tic is moderate or severe, behavioral intervention or drug intervention is required. Compared with drug therapy, only a limited number of children can use behavioral therapy [27]. Antidopaminergic drugs [28] (e.g., Haloperidol, pimozide, etc.) are widely used to treat children with tic disorders, but their side effects are obvious and the efficacy is poor. In China, TCM treatment of TD has shown good efficacy [29]. A variety of proprietary Chinese medicines have been developed, among which JWXFO has been proven to be a proprietary Chinese medicine that can significantly improve the status of symptoms of tic disorders in Chinese children, with few side effects [11–13].

The substitution of drugs with similar efficacy in traditional Chinese medicine prescriptions is the characteristic of clinical treatment of traditional Chinese medicine. The replacement of *Long Gu* (Os Draconis) by *Mu Li* (Concha Ostreae) with the same function is of great significance to reduce the damage to fossil resources and reduce the economic cost of treating children with TD. However, there is no relevant report on the clinical trial design of children replaced by traditional Chinese medicine. The research team reviewed the effectiveness data of ancient and modern substitutes for the two drugs and considered them equivalent after professional pharmacodynamic evaluation. Combined with the characteristics of traditional Chinese medicine, the clinical positioning and main evaluation indicators were determined through evidence-based research. After the high-level design meeting and feasibility study meeting convened by pediatricians, statisticians, methodology experts, and other multi-disciplinary experts, a clinical trial with the original preparation as the parallel control was finally developed. Compared with the traditional placebo control, it can significantly reduce the ethical risk of the clinical trial and improve the compliance of children and the operability of the trial. This trial plan is the first randomized controlled trial plan for the substitution of traditional Chinese medicine for children in clinical trials. Its randomized, double-blind trial design, which uses the original preparation as a control for equivalence test, will also provide reference for clinical trial design after the substitution of traditional Chinese medicine/herbal medicine at home and abroad.

## Trial status

Protocol version 1.0, 9 August 2019.The trial is currently in the process of recruiting participants in the thirteen study centers.

## Data Availability

The results will be published in a peer-reviewed journal. Datasets generated or analyzed in this study will be provided by the corresponding authors upon reasonable request.

## Abbreviations

TD: Tic disorders
JWXFD: Jiu-Wei-Xi-Feng granules (*Os Draconis* replaced by *Concha Ostreae*)
JWXFO: Jiu-Wei-Xi-Feng granules (original formula)
TCM: Traditional Chinese medicine
YGTSS-TTS: Yale Global Tic Severity Scale Total Tic Score
CGI-S: Clinical Global Impression-Severity scale
CRF: Case report form
AEs: Adverse events
CRC: Researchers or clinical research coordinator
FAS: Full analysis set
PPS: Per-protocol set
SS: Safety set
95%CI: 95% confidence interval
